# Cultivation of Microalgae and Cyanobacteria: Effect of Operating Conditions on Growth and Biomass Composition

**DOI:** 10.3390/molecules25122834

**Published:** 2020-06-19

**Authors:** Alejandra Sánchez-Bayo, Victoria Morales, Rosalía Rodríguez, Gemma Vicente, Luis Fernando Bautista

**Affiliations:** 1 Department of Chemical, Energy and Mechanical Technology, ESCET, Universidad Rey Juan Carlos, 28933 Móstoles, Madrid, Spain; alejandra.sanchezbayo@urjc.es (A.S.-B.); rosalia.rodriguez@urjc.es (R.R.); gemma.vicente@urjc.es (G.V.); 2 Department of Chemical and Environmental Technology, ESCET, Universidad Rey Juan Carlos, 28933 Móstoles, Madrid, Spain; victoria.morales@urjc.es

**Keywords:** microalgae, cyanobacteria, biomass composition, culture optimisation, growth on wastewaters

## Abstract

The purpose of this work is to define optimal growth conditions to maximise biomass for batch culture of the cyanobacterium *Arthrospira maxima* and the microalgae *Chlorella vulgaris, Isochrysis galbana* and *Nannochloropsis gaditana*. Thus, we study the effect of three variables on cell growth: i.e., inoculum:culture medium volume ratio (5:45, 10:40, 15:35 and 20:30 mL:mL), light:dark photoperiod (8:16, 12:12 and 16:8 h) and type of culture medium, including both synthetic media (Guillard’s F/2 and Walne’s) and wastewaters. The results showed that the initial inoculum:culture medium volume ratio, within the range 5:45 to 20:30, did not affect the amount of biomass at the end of the growth (14 days), whereas high (18 h) or low (6 h) number of hours of daily light was important for cell growth. The contribution of nutrients from different culture media could increase the growth rate of the different species. *A. maxima* was favoured in seawater enriched with Guillard’s F/2 as well as *C. vulgaris* and *N. gaditana,* but in freshwater medium. *I. galbana* had the greatest growth in the marine environment enriched with Walne’s media. Nitrogen was the limiting nutrient for growth at the end of the exponential phase of growth for *C. vulgaris* and *N. gaditana*, while iron was for *A. maxima* and *I. galbana*. The growth in different synthetic culture media also determines the biochemical composition of each of the microalgae. All species demonstrated their capability to grow in effluents from a wastewater treatment plant and they efficiently consume nitrogen, especially the three microalga species.

## 1. Introduction

Microalgae, such as cyanobacteria and eukaryotic algae, are photoautotrophic microorganisms that use inexpensive and widely available natural resources, such as CO_2_, H_2_O and inorganic salts, to transform radiant energy into valuable products contained in the biomass. These photosynthetic microorganisms are among the most promising new sources of energy, since they are renewable and neutral with respect to CO_2_ emissions. The CO_2_ emitted after their use corresponds to that fixed from atmosphere by microalgae, so that no net CO_2_ emission is produced. Although the number of different algae and cyanobacteria species in nature is unknown, conservative estimations give values above 70,000 of which more than 40,000 have been published [1]. Because of this enormous number of species, we selected four different microorganisms with different compositions and behaviour in this work.

*Arthrospira maxima* is a species belonging to the phylum of Cyanobacteria. These microorganisms are characterised for being able to perform photosynthesis and, traditionally, they have been grouped, alongside *A. platensis*, under the term Spirulina. Generally, they are arranged in multicellular filaments (50–500 µm long and diameters of 3–12 µm) and have been found in tropical and subtropical waters of alkaline lakes containing high concentrations of carbonate, either in salt or freshwater [2]. The main advantage of these species is their high protein content, therefore they are used as food supplements. They also present great benefits to human health due to their antioxidant properties, their role as activator of cell regeneration, and their positive effect on kidney and memory problems [3].

*Chlorella vulgaris* is a small, spherical microalga with a size of 5–10 μm, belonging to the family of Chlorellaceae. It has high protein content and a balanced amino acid composition, which means that it is widely employed for human feeding. Different studies also show the possible mixotrophic behaviour of this specie [4].

*Nannochloropsis gaditana* is a microalga with a small size, around 3 μm. This microalga belongs to the Eustigmataceae family and the name of the genus, *Nannochloropsis*, derives from its small size. The species of this genus usually grow in salt water, although they have also been found in fresh and brackish waters [5]. The main use of this alga is the feeding of fish in aquaculture due to the high accumulation of pigments, such as xanthophylls, and of polyunsaturated fatty acids [6]. However, in recent years, it has been used for the production of biofuels, such as biodiesel, due to its lipid accumulation potential [7].

The microalga *Isochrysis galbana* belongs to the family of Isochrysidaceae. It is a unicellular species with a brownish colour and it has two flagella, with sizes within the range 3 to 5 μm. *I. galbana* is known to have good nutritional qualities, and it is used in aquaculture as feed in the early larval stages of molluscs, fishes and crustaceans. It is characterised by its capability to accumulate polyunsaturated fatty acids, particularly omega 3 such as eicosapentaenoic (EPA) and docosahexaenoic (DHA). Currently, it is being used to study the immuno-modulation properties of some of its components [8].

Recently, these microorganisms have been studied to produce different biofuels such as biodiesel [9], bioethanol [10], biogas [11] and biohydrogen [12]. In this context, the study of the cultivation stage is crucial to obtain a suitable microalgal biomass for the production of biofuels. Thus, the growth of photosynthetic microalgae and cyanobacteria is governed by several factors, such as temperature, CO_2_ supply, pH, nutrients availability and light conditions, which should be optimised in photo-bioreactor systems for further industrial scale-up. Since light is the driving force of photosynthesis, it is the major abiotic parameter affecting cell metabolism. Photosynthetic organisms can be adapted to changes in light intensity and spectrum. Too much light may reduce microalgal productivity and have negative effects on the quality of biofuels, although photosynthetic organisms have evolved many strategies to protect themselves from photodamage. Light quality also has a substantial impact on cell metabolism [13], modulating the biomass composition of carbohydrates, lipids and proteins [14].

Photoperiod is a factor regulating cell division in asexual reproduction, which occurs during the light period, and it is accelerated under continuous illumination. Therefore, the photoperiod can be adjusted according to the objectives of the culture: a continuous lighting produces rapid growth, whilst a photoperiod, alternating hours of light and dark, like the solar photoperiod, maintains a normal and healthy growth. The short-term and long-term adaptations associated with balances in photosystem stoichiometry have been investigated in relation to the spectral characteristics of light [15].

It has been found that another key factor favouring microalga growth is the presence of micronutrients, such as vitamins, in the culture medium [16,17]. The axenic culture of microalgae demands a more stringent composition of nutrients in their cultivation medium. Therefore, special formulations of standard media such as Walne’s [18] and Guillard’s F/2 [19] media are normally used to produce microalga monoculture at the laboratory scale. The sufficient supplement of nutrients for microalga growth is a key step to produce a bulk quantity of high quality microalgal biomass. Microalgae can grow in polluted waters because the waters may contain some of the required nutrients. Therefore, municipal, industrial or agricultural wastewaters can be used as cultivation media while simultaneously act as a biological treatment to remove and recycle nitrogen and phosphorus from those streams [20,21,22].

In this work, we studied the effect of three key variables on the final maximum biomass growth of one cyanobacterium (*A. maxima*) and three microalgae (*C. vulgaris, N. gaditana* and *I. galbana*). First, we analysed the effect of the initial inoculum:culture medium volume ratio, the light photoperiod and the culture media composition for each microorganism. After studying the optimal culture conditions, the experiments were scaled-up to a larger volume (12 L) to proceed with the evaluation of the macromolecular and elemental composition of the microalgal biomass, measuring protein, carbohydrate and lipid content. Additionally, we studied the consumption of different nutrients and micronutrients during the growth and the results were evaluated in terms of dry weight and cell concentration fitting the results to Monod growth kinetics. With the aim to test growth and nutrients depletion in wastewaters, cultivation of microalgae and cyanobacteria was also carried out using water from different processes of a wastewater treatment plant.

## 2. Results and Discussions

### 2.1. Analysis of Variables to Maximize the Biomass

**Figure 1:**
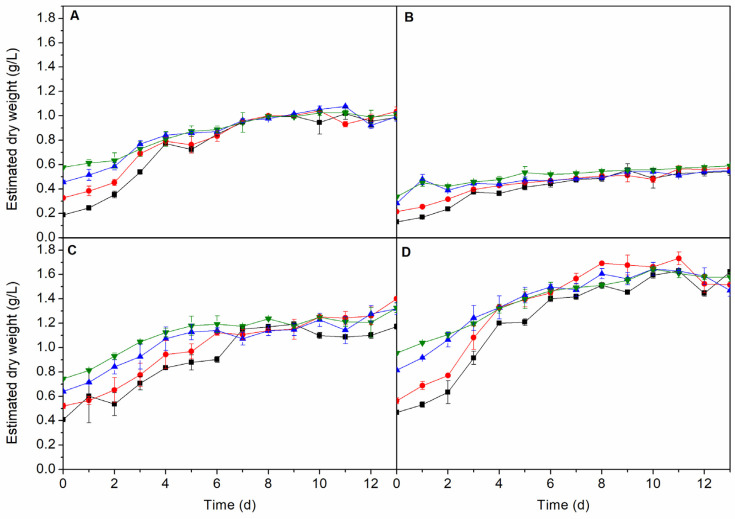
Growth of *A. maxima* (**A**), *C. vulgaris* (**B**), *I. galbana* (**C**) and *N. gaditana* (**D**) at different inoculum:culture medium ratio (■ 5:45, ● 10:40, ▲ 15:35 and ▼ 20:30) under 12:12 h light:dark photoperiod.

The inoculum:culture medium ratio was studied for all species at 25 °C using synthetic seawater with Guillard’s F/2 as a culture medium under 12:12 h light:dark photoperiod. As can be seen in Figure 1, there was no significant difference between the ratios studied at the stationary growth phase for each species. As cultures reach the end of the exponential phase, growth curves for different inoculum:culture medium ratios approach the same plateau value as observed for *I. galbana* cultivated in raceway ponds [8]. This may be due to the fact that biomass concentration was high enough to produce cell-shading.

Taking this into account, subsequent studies were performed using the lowest inoculum:culture medium ratio (5 mL inoculum:45 mL medium), since a low inoculum concentration decreases the costs without affecting the cellular reproduction time.

Light is a key parameter for the growth of photosynthetic organisms to carry out their metabolic processes [23,24]. The light:dark cycle determines the stage of development of the alga, i.e., cell division occurs during the light phase while the dark phase is used to accumulate reserves [25]. It has been shown that carbohydrates are mainly accumulated after light exposures above 8 h and, in the case of lipids, when the light period is longer than 12 h [26]. However, other studies have shown that the accumulation of lipids not only depends on the light phase, but also on other variables [27]. The results showed how the growth of *A. maxima*, *C. vulgaris* and *N. gaditana* depends largely on the number of hours of light exposure.

**Figure 2:**
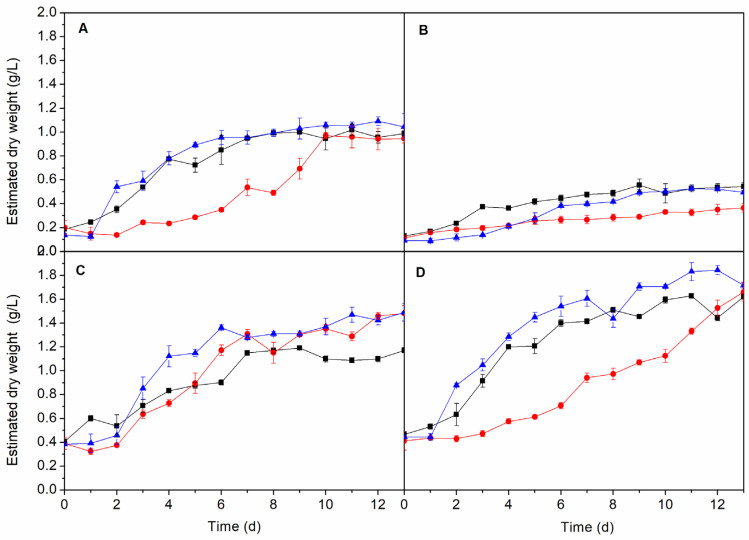
Growth of *A. maxima* (**A**), *C. vulgaris* (**B**), *I. galbana* (**C**) and *N. gaditana* (**D**) at different light:dark photoperiod (● 6:18, ■ 12:12 and ▲ 18:6 h).

The light:dark photoperiod was studied at three different values (Figure 2). The two photoperiods with a larger number of light hours (18:6 and 12:12) increased production of biomass with respect to the 6:18 photoperiod for *A. maxima*, *C. vulgaris* and *N. gaditana*. In the case of *A. maxima*, the amount of dry weight reached a maximum of 0.95 ± 0.03 g/L after eight days, regardless of the light cycle used (18:6 and 12:12) while the biomass concentration for the 6:18 light:dark photoperiod was significantly lower (0.54 ± 0.09 g/L, *p*-value > 0.05). However, for that cyanobacteria, there is a clear difference between the longest light cycle (18:6) and the shortest (6:18) where a long *lag* phase is observed in the latter. To a lesser extent, a similar behaviour was measured for *N. gaditana*, where an average biomass concentration of 1.5 ± 0.1 g/L was reached after eight days for 18:6 and 12:12 photoperiods, higher than 0.94 ± 0.05 g/L (*p*-value > 0.05), the value reached at that time when the 6:18 photoperiod was used. However, at the end of the cultivation (13 days) the average biomass concentration was 1.7 ± 0.1 g/L, not showing statistical differences between all three photoperiods. In the case of *C. vulgaris,* the shortest light cycle (6:18) did not show the exponential growth phase and longer exposures to light resulted in biomass concentration of approximately 0.5 ± 0.1 g/L at the end of the cultivation experiment. Growth of *I. galbana* seems to be somewhat insensitive to the light:dark photoperiod because, in all cycles studied, the stationary phase was reached after seven days attaining a dry weight of 1.2 ± 0.1 g/L independently of the photoperiod.

Therefore, with the objective of achieving the optimum conditions of culture for each species, a 12:12 photoperiod was selected for further studies, since it provides the larger amount of biomass in some cases and it is closer to the natural light–dark cycle. Therefore, by using the photoperiod at the industrial scale, the artificial supplementation of energy in the form of light is not necessary to reach the higher biomass concentration achieved in the laboratory.

Three synthetic marine culture media were tested for growing all four species (Table 1 and Table S1): Guillard’s F/2 (MC1), plain synthetic seawater (MC2) and Walne’s (MC3). Additionally, *A. maxima*, *C. vulgaris* and *N. gaditana* were grown in two freshwater culture media: Guillard’s F/2 (MC4) and Walne’s (MC5). All the experiments were conducted with an inoculum:culture medium ratio of 5:45 and a photoperiod of 12:12 h previously selected for optimal conditions.

**Figure 3:**
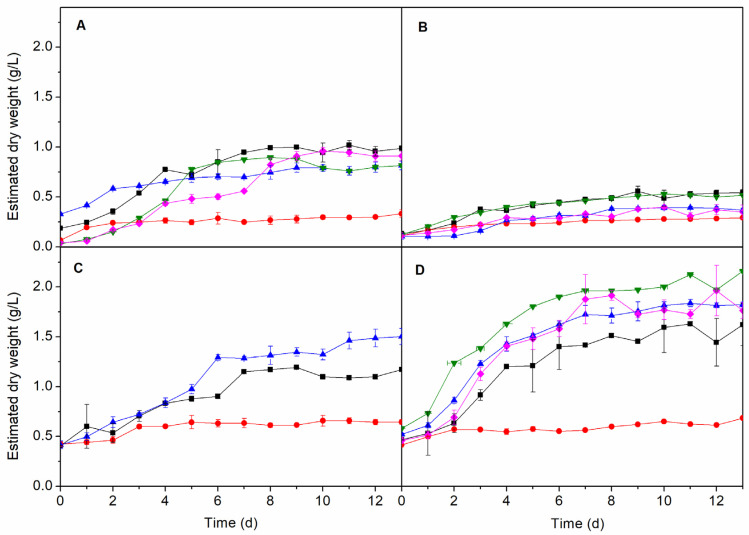
Growth of *A. maxima* (**A**), *C. vulgaris* (**B**), *I. galbana* (**C**) and *N. gaditana* (**D**) with different culture media (■ MC1, ● MC2, ▲ MC3, ▼ MC4 and ♦ MC5). Conditions for all cultivations: inoculum:culture medium ratio of 5:45 and a photoperiod of 12:12 h.

The results obtained with the synthetic marine water (MC2) for all microalgae (Figure 3) exhibit a very limited growth when compared to the other media, since MC2 is the only medium that was not enriched with vitamins and oligoelements. For *A. maxima*, the results showed that MC1 was the best culture medium (*p*-value < 0.05). Under these conditions, cell growth yielded 0.99 ± 0.02 g/L of dry biomass. For *I. galbana*, MC3 produced even higher biomass production (*p*-value < 0.05), reaching values of 1.50 ± 0.08 g/L of dry weight. The microalga growing in freshwater, i.e., *C. vulgaris* and *N. gaditana*, showed the best performance when cultured in MC4 medium, with biomass concentrations of 0.52 ± 0.03 and 2.16 ± 0.05 g/L, respectively, although MC1 produced similar growth on *C. vulgaris* (0.54 ± 0.03 g/L, *p*-value > 0.05).

From the above results, the optimal conditions for the growth of *A. maxima* and *I. galbana* are seawater enriched with Guillard’s F/2 (MC1) and Walne’s (MC3) media, respectively. On the other hand, *N. gaditana* and *C. vulgaris* showed their best performance in freshwater enriched with Guillard’s F/2 medium (MC4).

### 2.2. Nutrients Uptake

**Figure 4:**
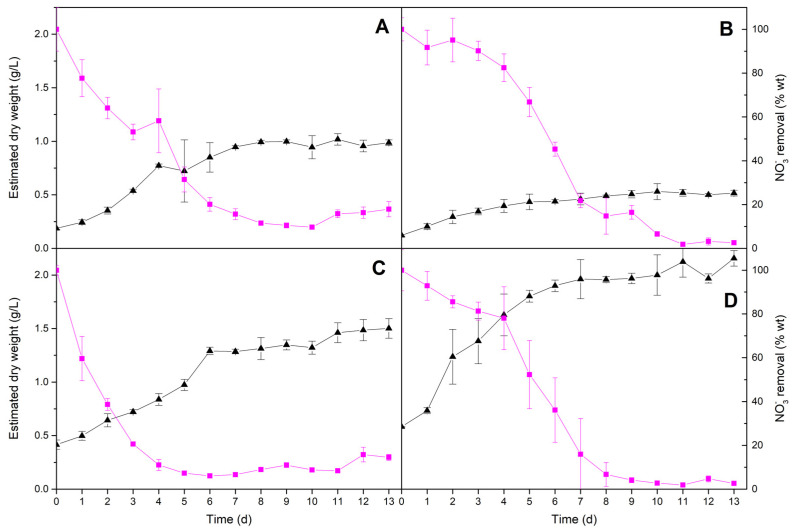
Biomass concentration (dry basis) (▲) and nitrate concentration in the culture medium (■) of *A. maxima* in MC1 (**A**), *C. vulgaris* in MC4 (**B**), *I. galbana* in MC3 (**C**) and *N. gaditana* in MC4 (**D**). Conditions for all cultivations: inoculum:culture medium ratio of 5:45 and a photoperiod of 12:12 h.

Cultivation media MC1 (for *A. maxima*) and MC4 (for *N. gaditana* and *C. vulgaris*) had an initial nitrate concentration of 134 mg/L and MC3 (for *I. galbana*) started with 100 mg/L of nitrate. Figure 4 shows nitrate removal by each microalga as growth progresses with time in cultivations performed under the optimal conditions selected previously. The results showed high nitrate removal (>97%) for *C. vulgaris* (97.4 ± 0.9%) and *N. gaditana* (97.4 ± 0.5%) by the end of the experiments. In the case of the cultivation performed with *I. galbana*, nitrate was depleted in a 93.9 ± 0.3% by day six and for *A. maxima*, the maximum nitrate removal reached 90.3 ± 0.5% at day 10. The above values agree with other results found in the literature [28,29]. In the individually optimised culture conditions, *C. vulgaris* showed higher nitrogen requirements for growth because that species consumed most nitrogen to yield the lowest (*p*-value < 0.05) biomass concentration (0.52 ± 0.03 g/L) at the end of the experiment (Figure 4B) as evidenced by its lower (*p*-value < 0.05) biomass yield value (Y_X/S_ = 3.0 ± 0.2 g/g, Table 2). Meanwhile, *N. gaditana* was able to grow up to 2.15 ± 0.07 g/L (Figure 4D), more than two-fold to that observed for *A. maxima*, 0.99 ± 0.03 mg/L (Figure 4A) showing similar nitrogen removal in both cases. Therefore, nitrogen is an essential nutrient acting as a possible limiting substrate in the cultivation. The nitrogen/phosphorus ratio for the used nutrient medium based on Guillard’s F/2 and Walne’s were 44.4 and 7.5, respectively, which correspond to common values reported in literature where nitrogen proved to be the limiting nutrient [30].

From the exponential growth phase and nitrate consumption (Figure 4), the Monod parameters (µ_max_ and K_S_) were calculated for all four species (Table 2) using Equations (1) and (2) (see Section 3.5. Growth kinetics).

The growth kinetic parameters can be notably affected by culture conditions, including ambient conditions and type and concentration of nutrients [31]. Although the values of the maximum specific growth rate in similar experimental conditions have not been reported for *A. maxima*, the value achieved in this work (µ_max_ = 0.202 ± 0.006 d^−1^) was higher (*p*-value < 0.05) than that found for other *Arthrospira* species (*A. platensis*) cultivated in batch mode using a medium with similar salinity (µ_max_ = 0.182 ± 0.007 d^−1^) [32]. For *C. vulgaris*, the value of µ_max_ = 0.27 ± 0.02 d^−1^ was within the range (0.25–0.32 d^−1^) of other studies where this species was cultured in wastewaters supplemented with ammonium [11]. Similar values were measured for the microalgae *I. galbana* (µ_max_ = 0.145 ± 0.003 d^−1^) and *N. gaditana* (µ_max_ = 0.181 ± 0.008 d^−1^). With respect to the half-saturation parameter, the K_s_ values showed a high affinity of each microorganism for nitrate, especially in the case of *A. maxima* and *N. gaditana*, whose low values were 1.7 ± 0.8 mg/L and 4 ± 2 mg/L, respectively. This means that both species can be very efficient at depleting nitrates from culture media up to very low concentrations. For *C. vulgaris* (K_S_ = 38 ± 9 mg/L) and *I. galbana* (K_s_ = 11.8 ± 0.7 mg/L), the half-saturation constants, although higher than those measured for the other two studied species, are low enough to achieve a good nitrate removal efficiency, showing similar values than those reported in the literature for nitrate as limiting nutrient [21].

**Figure 5:**
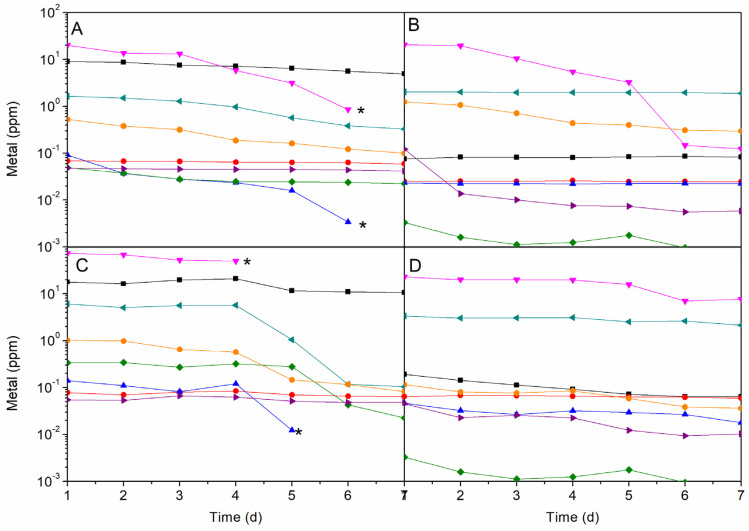
Trace element concentration in the culture media during optimum growth of *A. maxima* in MC1 (**A**), *C. vulgaris* in MC4 (**B**), *I. galbana* MC3 (**C**) and *N. gaditana* in MC4 (**D**) (■ B, ● Co, ▲ Cu, ▼ Fe, ♦ Mn, ◄ Mo, ► P, ● Zn, * completely consumed). Conditions for all cultivations: inoculum:culture medium ratio of 5:45 and a photoperiod of 12:12 h.

To evaluate other essential nutrients, the consumption of oligoelements was monitored in the culture media for seven days (Figure 5). All microalgae consumed all iron present in the culture media in a few days. *I. galbana* needed a larger amount of iron (70 mg/L) compared with the rest of the species (approximately 20 mg/L). In the case of microalgae cultivated in freshwater, a notable consumption of iron was observed, with 28% and 12% of iron remaining unused in the case *C. vulgaris* and *N. gaditana*, respectively. However, these microalgae have a total consumption of nitrate leaving some iron present in the culture media. Marine microalgae completely consumed copper, whereas in the case of freshwater species it was not significantly depleted. The presence of boron in marine species also decreased.

### 2.3. Biomass Composition

At the optimum conditions selected for each species, growth was studied during 14 days in batch cultures, monitoring pH, biomass composition and nutrient uptake. The pH is determinant for microalgal growth and all initial culture media had a pH value of 7.5. During cultivation, all species increased their pH over time, but this increase was mainly concentrated in the first part of the exponential phase (data not shown). The values reached for *A. maxima* were greater than 8, a value that corresponds to the optimal values of the species of the genus *Arthrospira*, which generally develop in alkaline media [33]. The culture carried out with *C. vulgaris* in fresh culture medium generated a pH increase from the initial one of 7.5 to 9 during the first 48 h. This is associated with the fast CO_2_ consumption driving the pH towards alkaline values. This microalga is grown in a wider pH range than the rest of the microalgae (6.3–9) [34]. *N. gaditana* is a species capable of developing at high pH values, around 8–10. The values obtained along the growth, around 8.5, were similar to those found in the literature [35,36], confirming that the medium used provided a pH environment suitable for growth. In the case of *I. galbana*, pH values obtained were slightly higher than the rest of the species, being around pH 9 [37].

The final pH was within the range 8–9 in all experiments, consistent with the values found in the literature [35], confirming that the media used provide a suitable pH environment for growth. The cultivation under extreme pH conditions, both acidic and basic, could allow for the use of certain wastewater for the growth of the four species studied [38].

**Figure 6:**
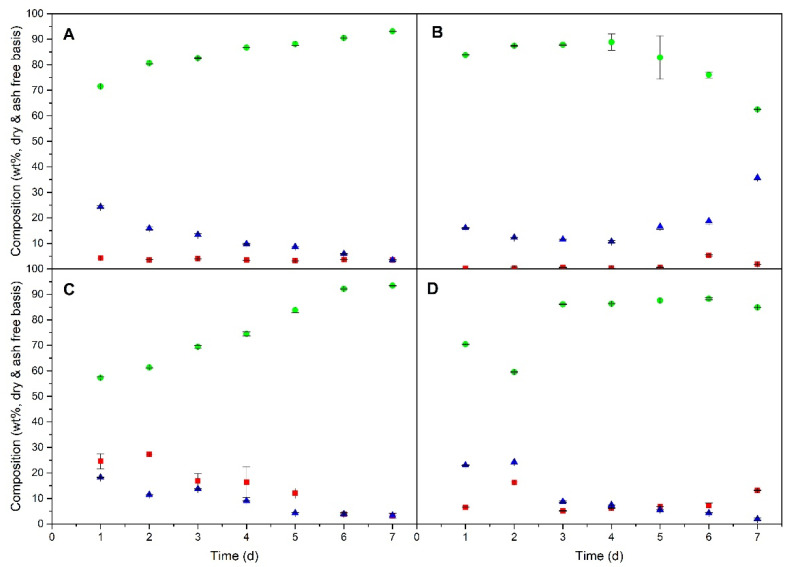
Time series of biomass composition (wt%, dry and ash free basis) of: ■ lipids, ● proteins and ▲ carbohydrates) for *A. maxima* in MC1 (**A**), *C. vulgaris* in MC4 (**B**), *I. galbana* in MC3 (**C**) and *N. gaditana* in MC4 (**D**). Conditions for all cultivations: inoculum:culture medium ratio of 5:45 and a photoperiod of 12:12 h.

The major building blocks of biomass from microalgae and cyanobacteria are proteins, carbohydrates and lipids, which were analysed until the end of the exponential phase of growth (first seven days of the trial). Figure 6 shows the monitoring of the biochemical composition during microbial growth at the optimum conditions selected for each species so far.

Protein content increased during the exponential growth phase for *A. maxima* (Figure 6A) and *I. galbana* (Figure 6C), reaching values of 93.1 ± 0.1% and 93.4 ± 0.1%, respectively, in line with previous studies [39]. However, the accumulation of proteins attained a maximum before the exponential phase ended for *C. vulgaris* (Figure 6B) and *N. gaditana* (Figure 6D). The protein concentration started to decline after four days in *C. vulgaris*. The protein content reached by *I. galbana* (93.4 ± 0.1%) was higher than that reported in Guillard’s F/2 under continuous irradiance (45.31%) [40]. This highlights the effect of the photoperiod on the composition of cell biomass. Of all protein content, soluble proteins were a small fraction of around 1–3%, following a similar trend to that observed for total protein accumulation (data not shown).

Carbohydrates in *A. maxima* (Figure 6A), *I. galbana* (Figure 6C) and *N. gaditana* (Figure 6D) showed decreasing values, from 15–25% to around 2–4%, as growth progressed. This tendency is in agreement with other results shown in the literature [40,41,42]. However, carbohydrate content in *C. vulgaris* (Figure 6B) was approximately constant during the exponential phase, with values ranging from 10–18%. Other studies [41], where low-nitrate modified Zarrouk’s medium [43] was used, described higher carbohydrate accumulation in spirulina species than those reached by *A. maxima* in the present work. This higher amount of carbohydrates can be explained by the larger nitrate limitation and the greater availability of carbon due to the presence of HCO_3_^-^ in Zarrouk’s medium [44]. Most carbohydrates are not soluble and, thus, must be complex since carbohydrates mainly form cell walls as cellulose-like polymers and chitin-like glycans [45]. The lipid content during the first few days of cultivation (Figure 6) was lower than the values usually reported as the maximum lipid concentration for these species because the synthesis and accumulation of lipids in microalgae and cyanobacteria increases at the end of the exponential growth phase, when nitrogen starvation takes place (see, for example, [46,47,48]).

To contribute to a deeper understanding of biomass composition, elemental analysis was also performed during the lag and the exponential phases of growth of all four species. Therefore, the amounts (wt%, dry basis) of carbon, hydrogen, nitrogen, sulfur and oxygen were determined (Tables S6 and S5). The carbon contents were high (20–53%) and the nitrogen and hydrogen contents were minor as expected (2–6.75% and 4–8%, respectively). However, sulfur content was low (<3.5%) and corresponds to sulfur amino acids (methionine and cysteine) in proteins.

One of the most important elements of study in the case of microalgae is the presence of nitrogen. Nitrogen is mainly contained in proteins and, to a lesser extent, in glycolipids from the cell walls and membranes, amino acids and sphingolipids. The nitrogen content was, in general, low (2–6%), showing the lowest values in the case of *A. maxima* and *I. galbana*. This can be associated with lower protein content in these biomasses. As a result of the elemental analysis (see Tables S2–S5), the following average molecular formulae, in the exponential phase, were obtained for each species: CH_1.93_O_1.61_N_0.11_S_0.02_ (*A. maxima*), CH_1.71_O_0.79_N_0.07_S_0.01_ (*C. vulgaris*), CH_2.73_O_1.09_N_0.09_S_0.04_ (*I. galbana*) and CH_1.61_O_0.60_N_0.12_S_0.01_ (*N. gaditana*).

### 2.4. Growth in Wastewaters

**Figure 7:**
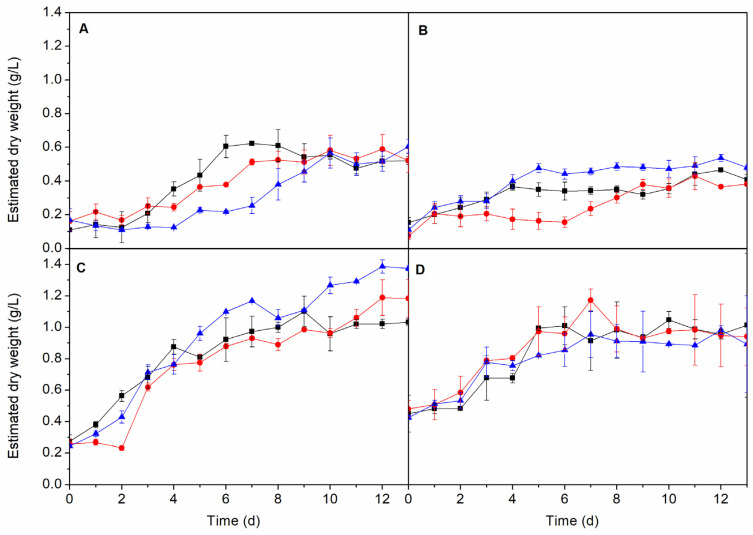
Growth of *A. maxima* (**A**), *C. vulgaris* (**B**), *I. galbana* (**C**) and *N. gaditana* (**D**) with different wastewater (■ AD1, ● AD2, ▲ AD3). Conditions for all cultivations: inoculum:culture medium ratio of 5:45 and a photoperiod of 12:12 h.

All microalgae and cyanobacterium studied were able to grow in wastewaters (Table 1) maintaining the inoculum:culture medium ratio of 5:45 and a light photoperiod of 12:12, obtaining similar biomass production in the three non-sterilised wastewater samples (AD1, AD2 and AD3) (Figure 7). The above-four species were capable of developing in non-specific media as long as they had a certain content of salt in the culture medium that promoted metabolic development. This is due to the metals present in wastewater that serve as essential trace elements for the development of these species.

To evaluate the consumption of nutrients from wastewater, the analysis of the cultures performed in AD2 was carried out (see Figure S1), because the growth curves were similar in media AD1, AD2 and AD3. Generally, most of the nitrogen originating from sewage water is in the form of ammonium, nitrite or nitrates [49]. Initially, it is usually found that ammonium is converted into nitrite and then nitrate after a conventional biological treatment in the presence of nitrifying bacteria in a period of 24 h, or it can be consumed in the form of ammonium [50,51,52]. Once the nitrogen is in the form of nitrate, it can be uptaken by the microalgae to proceed to the bioremediation of the water [53]. The nitrate content of the water sample at the outlet of the separator after the biological treatment (AD2) was 6.9 ppm (Table S6). This low level of nitrate concentration may be due to the fact that in the biological separator certain bacteria were fed by this nutrient. The results showed a significant decrease of nitrates with microalgae *C. vulgaris*, *I. galbana* and *N. gaditana* up to 1–2 ppm, thus consuming 70–80% of the total. On the contrary, in the case of cyanobacterium, *A. maxima* only reached a consumption of about 30%. This low nitrate depletion efficiency may be due to the need of a photoperiod with more light hours to increase the assimilation or the lack of phosphorus in the culture medium [54,55].

Despite the low concentration of nitrates in the culture medium, their low uptake can be caused by another limiting nutrient. There is a clear decrease in phosphorus in the culture medium (>90%), for *A. maxima* and *I. galbana* while the rest of the elements present in the medium were consumed to a lesser extent (Table S7). This may be due to the fact that these metals are needed in very low amounts for the metabolic development of these species. All this seems to indicate that in these wastewaters, both nitrates and phosphorus, as well as the light photoperiod, must keep an optimum ratio to achieve a complete depletion of both nutrients [56,57].

## 3. Materials and Methods

### 3.1. Microorganism

*Arthrospira maxima* (SAG 49.88), *Chlorella vulgaris* (SAG 211.11-b), *Isochrysis galbana* (SAG 13.92) and *Nannochloropsis gaditana* (SAG 2.99) strains were obtained from the SAG Culture Collection of Algae (University of Gottingen, Gottingen, Germany).

### 3.2. Chemicals

Standards d-glucose (>99.5% purity) and sulfanilic acid (>99% purity) were purchased from Sigma-Aldrich (Saint Louis, MO, USA), sodium nitrate (>99% purity) from Scharlab (Barcelona, Spain) and bovine serum albumin (Quick Start BSA) from Bio-Rad Laboratories (Hercules, CA, USA). All other chemicals used were reagent grade: ethylenedinitrilotetraacetic acid disodium salt, phenylmethylsulfonyl fluoride, Triton-X100, dinitrosalicylic acid, sodium potassium tartrate tetrahydrate and methanol were supplied by Sigma-Aldrich and chloroform, sodium hydroxide, 98% sulfuric acid and 48% hydrofluoric acid were purchased from Sharlab.

### 3.3. Growth Experiments

Microalgae cultivations were carried out in 50 mL batch mini-photobioreactors where atmospheric air was continuously bubbling and in-line sterilised through a 0.45 μm membrane filter. Air flow was provided only during light periods at a rate of 1.75 L/min. The inoculum:culture medium volume ratio used in this work were 5:45, 10:40, 15:45 and 20:30 mL:mL in order to assess whether a larger number of initial cells affects the growth rate and total biomass production at the end of the experiment or not. These ratios were chosen based on previous studies [16,39,58,59,60]. We tested three different light:dark photoperiods, i.e., 6:18 h, 12:12 h and 18:6 h, using discontinuous artificial illumination provided by 12 V/24 W white LED lamps under an irradiance value of 108 µmol·photon·m^−2^·s^−1^.

Each species was cultivated in two different synthetic nutrient media: Guillard’s F/2 (EasyAlgae. Cádiz, Spain) and Walne’s (Aqualgae. A Coruña, Spain), both in fresh and seawater. Synthetic seawater was produced with CoralMarine EasyMix (GroTech GmbH. Langenhansen, Germany) according to manufacturer’s instructions, to a final concentration of 33.3 g/L. Microalgae were also cultivated in waters withdrawn from different stages of Rey Juan Carlos University waste water treatment plant located in its Campus in Mostoles (Madrid, Spain). Table 1 shows the identification of each cultivation media and the composition of media and wastewaters is shown in Tables S1 and S6, respectively.

The microalgae and cyanobacteria were allowed to grow for two weeks at 25 °C. The optical density of the culture was measured using a V-630 spectrophotometer (Jasco Analítica Spain, Madrid, Spain) at 540 nm. To determine the dry weight content, a calibration with biomass from the stationary phase was performed to establish the absorbance-dry weight relationship. For this purpose, a homogeneous sample of the culture was centrifuged at 5000 rpm for 5 min and then the solid phase containing biomass was dried at 105 °C overnight and allowed to cool down to room temperature inside a desiccator to obtain the weight of dry microalgae. The pH was measured every day with a Basic 30 pH meter (Crison Instruments. Barcelona, Spain).

Once the culture conditions had been optimised in the mini-photobioreactors, the process was scaled to a 12 L photobioreactor to fully characterise the microalgal biomass as a function of time and state of growth of the microalgae. A modified Bioflo 110 (New Brunswick Scientific, Edison, NJ, USA) bioreactor/fermenter, adapted for the culture of photosynthetic microorganisms, was used as a photobioreactor system. An in-house designed 12 L jacketed glass vessel was employed, in which pH, temperature and stirring were controlled automatically. The culture vessel was placed inside a metal housing fitted with 8 W Sylvania fluorescent lights, which allow controlling the lighting inside the reactor during the chosen light photoperiod. Aeration of the reactor was carried out by a membrane pump at a flow rate of 1.75 L/h, providing the necessary CO_2_ amount as carbon source.

### 3.4. Analysis of Biomass

Every day, 0.5 L of culture was withdrawn, centrifuged and vacuum-dried to obtain the biomass for further analysis. Dry microalga cells (0.5 g) were lysed with 5 mL lysis buffer (aqueous solution containing 1.1 mM ethylenedinitrilotetraacetic acid disodium salt, 0.2 mM phenylmethylsulfonyl fluoride and 0.5% Triton-X100) under vortex stirring for 5 min. Protein concentration was measured by Bradford’s method [61] using Quick-Start^®^ Protein Assay Kit (Bio-Rad Laboratories. Hercules, CA, USA) according to manufacturer’s instructions. The method is based on the shift of the wavelength of maximum absorbance of the dye reagent Coomassie^®^ Brilliant Blue G-250 from 465 nm to 595 nm when it is bonded to a protein. The quantification of protein concentration was performed by measuring the absorbance at 595 nm in a V-630 spectrophotometer (Jasco Analítica. Madrid, Spain) using bovine serum albumin as standard protein to build the calibration curve assuming Lambert–Beer law is followed.

The carbohydrate content was measured by the DuBois method [62] using glucose as calibration standard. The carbohydrate soluble fraction was measured from lysed cells suspension using a DNS solution (1 g of 3,5-dinitrosalicylic acid, 30 g of sodium potassium tartrate tetrahydrate, 50 mL of water and 20 mL 2N NaOH). The procedure was as follows: 1 mL of each sample and each calibration solution was placed in a test tube. Then, 1 mL of DNS was added, and the resulting solution was vortexed, heated in a water bath at 100 °C for 10 min and cooled by quenching in ice. Finally, 10 mL of distilled water were added, and the solution was measured with a V-630 spectrophotometer at 540 nm.

Elemental analysis was performed in a Vario EL III CHNS (Elementar Analysensysteme GmbH. Langenselbold, Germany) to quantify the amount of carbon, hydrogen, nitrogen, and sulfur in the microalgae and the oxygen amount was calculated by mass balance. Around 10 mg of dry weight were weighted and fully oxidised in an air stream at 1000 °C. Then, the oxidation products were transported by a carrier gas (helium) through selective adsorption columns. The gases were then measured with a thermal conductivity detector. Sulfanilic acid was used as standard for element quantification.

The lipids were extracted using a chloroform:methanol (4/5 *v/v*) solvent mixture and the lipid concentration was calculated gravimetrically [63]. Nitrate content was measured using a 781 pH/Ion meter equipped with a polymer–membrane selective electrode (Metrohm. Herisau, Switzerland). For the quantification, a standard calibration curve with sodium nitrate was prepared.

The metal content in the microalgal biomass and in the solution of the cultivation media was monitored by induced plasma atomic emission spectroscopy (ICP-AES) on a Vista AX CCD simultaneous ICP-AES (Varian. Palo Alto, CA, USA). Dry microalgae biomass (0.1 g) were treated by acid digestion with 2 mL of 98% sulfuric acid and 10 mL of deionised water. The sample was heated to dryness. The resulting solid was calcined at 750 °C (heating at 50 °C/min) for 5 h. The resulting ash was digested with 2 mL of 98% sulfuric acid and 10 mL of 48% hydrofluoric acid and heating until complete evaporation of hydrofluoric acid. Then, the liquid sample was diluted with deionised water up to 50 mL for analysis. Filtration through a 0.45 µm nylon membrane was the only preparation treatment for the samples of aqueous cultivation media.

All analysis were performed in triplicate to allow calculation of average values and standard errors of each measurement.

### 3.5. Growth Kinetics

Biomass growth and nutrient removal was monitored as described above and that information was used to model the specific growth rate (*µ*) following a first-order kinetics (Equation (1)) assuming a Monod-type kinetics with nitrate as limiting nutrient (Equation (2)).

(1) $$\mu =\frac{1}{X}\frac{dX}{dt}$$

(2) $$\mu =\mu_{\max}\frac{S}{K_{S}+S}$$

where *X* is the biomass concentration in dry basis (g/L), *S* the nitrate concentration (mg/L), *µ*_max_ the maximum specific growth rate (d^−1^) and *K_S_* the half-saturation constant (mg/L). Therefore, the kinetic parameters µmax and *K_S_* can be calculated from the monitoring of growth and nitrate consumption with time during the exponential phase, assuming that the specific growth rate is constant during this phase. Growth and nitrate uptake are also related through the corresponding yield coefficient (*Y_X/S_*, Equation (3)) which gives the amount of biomass produced by unit mass of nitrate consumed by the microorganism.

(3) $$Y_{X/S}=-\frac{\Delta X}{\Delta S}$$

### 3.6. Statistical Analysis

Numerical experimental results are shown as the mean value of three replicates ± standard deviation. One-way Analysis of Variance (ANOVA) was performed using Statgraphics Centurion XVII (StatPoint Technologies, Inc. Warrenton, VA, USA) to assess the statistical significance of the means between different treatments. The criteria to consider statistical significance between different means was *p*-value < 0.05.

## 4. Conclusions

The effect of cultivation variables on the growth of three microalgae and one cyanobacterium with potential industrial interest was evaluated. Whereas the initial inoculum volume did not affect the final biomass production, the amount of light to which the microalgae were exposed was a significant factor. Thus, the photoperiod 12:12 h light:dark was the most favourable for the growth of the four species. The analysis of biomass composition showed that for *A. maxima* and *I. galbana*, the protein content increased during the exponential phase while for *N. gaditana* and *C. vulgaris*, the accumulation of proteins ended before the stationary phase was reached. Nitrate and iron, as macro- and micro-nutrients respectively, became the limiting compounds for all four species, with copper being essential for growth in marine media. Although all of the species studied were able to grow in wastewaters, *I. galbana*, *N. gaditana* and *C. vulgaris* achieved higher nitrogen removal capacity than cyanobacterium *A. maxima*.

## Figures and Tables

**Table 1:** Cultivation media ^1^.

Identification	Type	Description
MC1	Synthetic seawater	Guillard’s F/2 medium
MC2	Synthetic seawater	Distilled water with 33 g/L of marine salt with 50 mg/L Mg and 400 mg/L Ca.
MC3	Synthetic seawater	Walne’s medium
MC4	Freshwater	Guillard’s F/2 medium
MC5	Freshwater	Walne’s medium
AD1	Wastewater	After primary decanter
AD2	Wastewater	After biological treatment
AD3	Wastewater	After secondary decanter

^1^ Composition of all cultivation media are shown in Table S1 (Supplementary Materials).

**Table 2:** Monod’s kinetic parameters.

Species	µ_max_ (d^−1^) ^1^	K_S_ (mg/L) ^2^	Y_X/S_ (g/g) ^3^
*A. maxima*	0.202 ± 0.006	1.7 ± 0.8	6.7 ± 0.2
*C. vulgaris*	0.27 ± 0.02	38 ± 10	3.0 ± 0.2
*I. galbana*	0.145 ± 0.003	11.8 ± 0.7	12 ± 1
*N. gaditana*	0.181 ± 0.008	4 ± 2	12.0 ± 0.6

^1^ Maximum specific growth rate, ^2^ Half-saturation constant, ^3^ Biomass yield on nitrate. Common conditions for all cultivations: inoculum:culture medium ratio of 5:45 and a photoperiod of 12:12 h. Growth media: *A. maxima* (MC1), *C. vulgaris* (MC4), *I. galbana* (MC3) and *N. gaditana* (MC4).

## Supplementary Materials

The following are available online, Table S1. Composition of Guillard’s F/2 and Walne’s media, Table S2: Elemental composition (%) of *A. maxima* at different times, Table S3: Elemental composition (%) of *C. vulgaris* at different times, Table S4: Elemental composition (%) of *I. galbana* at different times, Table S5: Elemental composition (%) of *N. gaditana* at different times, Table S6: Characterisation of water samples from wastewater treatment plant located at Campus of Móstoles (Universidad Rey Juan Carlos), Table S7: Phosphorus depletion in wastewater AD2, Figure S1: Nitrate uptake in wastewater AD2.
