# Supplementary material for: Cultivation of Microalgae and Cyanobacteria: Effect of Operating Conditions on Growth and Biomass Composition
Source: Molecules. 2020 Jun 19;25(12):2834. doi: 10.3390/molecules25122834 (PMC7356364; doi:10.3390/molecules25122834)
Supplement: Supplementary file 1 [file molecules-25-02834-s001.pdf]

<u>Supplementary material</u>

Cultivation of microalgae and cyanobacteria: Effect of operating conditions on growth and biomass composition

Alejandra Sánchez-Bayo [1], Victoria Morales [2], Rosalía Rodríguez [1], Gemma Vicente [1], Luis Fernando Bautista [2,*]

[1] Department of Chemical, Energy and Mechanical Technology, ESCET, Universidad Rey Juan Carlos. C/ Tulipán s/n. 28933 Móstoles, Madrid, Spain.
[2] Department of Chemical and Environmental Technology, ESCET, Universidad Rey Juan Carlos. C/ Tulipán s/n. 28933 Móstoles, Madrid, Spain.

[*] Corresponding author:
Luis Fernando Bautista
E-mail: fernando.bautista@urjc.es

Table S1. Composition of Guillard's F/2 and Walne's media.

Guillard's F/2 [1]		Walne's [2]	
Compound	Concentration (g/L)	Compound	Concentration (g/L)
$CoCl_2{\cdot}6\ H_2O$	$1.00{\cdot}10^{-4}$	$ZnCl_2$	$2.10{\cdot}10^{-4}$
$Na_2Mo_7O_4{\cdot}2H_2O$	$6.00{\cdot}10^{-6}$	$CoCl{\cdot}6\ H_2O$	$2.00{\cdot}10^{-7}$
$FeCl_3{\cdot}6H_2O$	$3.00{\cdot}10^{-3}$	$(NH_4)_6Mo_7O_{24}{\cdot}4H_2O$	$9.00{\cdot}10^{-8}$
$MnCl_2{\cdot}4H_2O$	$1.80{\cdot}10^{-4}$	$CuSO_4{\cdot}5H_2O$	$2.00{\cdot}10^{-7}$
Na_2EDTA	$4.65{\cdot}10^{-3}$	$FeCl_3{\cdot}6H_2O$	$1.30{\cdot}10^{-3}$
$NaH_2PO_4{\cdot}2H_2O$	$5.65{\cdot}10^{-3}$	$MnCl_2{\cdot}4H_2O$	$3.60{\cdot}10^{-4}$
$NaNO_3$	$1.34{\cdot}10^{-1}$	H_2BO_3	$3.36{\cdot}10^{-2}$
$CuSO_4$	$1.00{\cdot}10^{-5}$	EDTA	$4.50{\cdot}10^{-2}$
$ZnSO_4$	$2.20{\cdot}10^{-5}$	$NaH_2PO_4{\cdot}2H_2O$	$2.50{\cdot}10^{-2}$
Vitamin B_{12}	$5.00{\cdot}10^{-7}$	$NaNO_3$	$1.00{\cdot}10^{-1}$
Vitamin B_1	$1.00{\cdot}10^{-4}$	TMS	$1.00{\cdot}10^{-3}$
Vitamin H	$5.00{\cdot}10^{-6}$	Vitamin B_{12}	$1.00{\cdot}10^{-4}$
		Vitamin B_1	$1.00{\cdot}10^{-4}$
		Vitamin H	$2.00{\cdot}10^{-4}$

[1] Salinity: 0.1 g/L; [2] Salinity: 0.2 g/L

Table S2: Elemental composition (%wt) of *A. maxima* at different times (Growth conditions: 5:45 inoculum:culture medium ratio, 12:12 h light:dark photoperiod and MC1 medium).

Time (d)	C (%)	H (%)	O (%)	N (%)	S (%)
1	32±2	5.2±0.3	58±2	3.0±0.2	1.1±0.3
2	31±2	4.9±0.2	60±2	3.4±0.2	1.15±0.09
3	19.5±0.1	2.8±0.4	74.4±0.3	2.07±0.04	1.2±0.2
4	30±1	4.8±0.2	59±2	4.3±0.2	1.7±0.2
5	26.8±0.4	4.41±0.04	63.2±0.4	3.95±0.04	1.59±0.08
6	23.3±0.5	4.1±0.1	68.1±0.7	3.2±0.1	1.34±0.01
7	19±1	3.6±0.2	73±1	2.5±0.2	1.8±0.3

Table S3: Elemental composition (%wt) of *C. vulgaris* at different times (Growth conditions: 5:45 inoculum:culture medium ratio, 12:12 h light:dark photoperiod and MC4 medium).

Time (d)	C (%)	H (%)	O (%)	N (%)	S (%)
1	7±1	1.9±0.2	88±2	1.1±0.1	2.1±0.3
2	22±1	1.0±0.1	70±1	3.3±0.1	3.49±0.09
3	28±1	0.54±0.02	63±1	3.6±0.1	4.6±0.1
4	47±2	7.6±0.3	40±2	4.2±0.2	0.70±0.06
5	49±3	8.2±0.4	38.9±0.4	3.4±0.2	0.52±0.02
6	53±2	8.56±0.03	34±3	3.28±0.07	0.63±0.09
7	47.86±0.08	7.77±0.2	35.64±0.08	7.93±0.02	0.8±0.2

Table S4: Elemental composition (%wt) of *I. galbana* at different times (Growth conditions: 5:45 inoculum:culture medium ratio, 12:12 h light:dark photoperiod and MC3 medium).

Time (d)	C (%)	H (%)	O (%)	N (%)	S (%)
1	43±2	6.7±0.3	48±2	1.908±0.001	0.72±0.05
2	37.56±0.04	5.80±0.06	53.0±0.2	2.66±0.01	0.96±0.04
3	30.9±0.1	4.47±0.08	60.6±0.4	3.22±0.08	0.83±0.05
4	27.1±0.1	5.07±0.04	62.1±0.5	3.17±0.02	2.54±0.06
5	19.5±0.7	3.2±0.6	73±2	2.3±0.7	2.44±0.07
6	20±1	3.9±0.3	71±3	2.7±0.4	1.8±0.1
7	16±1	3.3±0.2	76±1	2.3±0.3	2.0±0.1

Table S5: Elemental composition (%wt) of *N. gaditana* at different times (Growth conditions: 5:45 inoculum:culture medium ratio, 12:12 h light:dark photoperiod and MC4 medium).

Time (d)	C (%)	H (%)	O (%)	N (%)	S (%)
1	47.1±0.2	6.92±0.09	38.9±0.5	6.4±0.1	0.60±0.20
2	45.5±0.8	6.6±0.2	40.7±0.9	6.47±0.05	0.64±0.04
3	45.42±0.03	6.75±0.01	40.80±0.01	6.43±0.01	0.59±0.01
4	43.4±0.03	6.25±0.01	42.97±0.03	6.75±0.05	0.64±0.01
5	46.5±0.4	6.74±0.07	39.9±0.2	6.2±0.1	0.58±0.01
6	46.4±0.05	6.64±0.07	40.0±0.6	6.39±0.07	0.58±0.01
7	46.9±0.9	6.9±0.1	39±1	6.55±0.06	0.65±0.02

Table S6: Characterisation of water samples from wastewater treatment plant located at Campus of Móstoles (Universidad Rey Juan Carlos).

	AD1	AD2	AD3
Total hardness (mg/L)	250	200	210
Chloride (ppm)	280	166	130
BOD (mg/L)	200	50	0-5
COD (mg/L)	700	200	150
Total solids at 105ºC (mg/L)	0.8	0.66	0.5
Total solids 155ºC (mg/L)	0.44	0.4	0.4
Conductivity (µS/cm)	1600	940	750
Turbidity	180	12	4
pH	7.06	7.34	7.41
[NO_3^-] (ppm)	3.5	6.9	59.41
TOC	150	35	20
Elements (ppm)			
B	0.106	0.246	0.018
Co	$6.11 \cdot 10^{-4}$	0	0
Cu	0.042	0.012	0.013
Fe	0.100	0.163	0.159
K	36.120	25.644	18.406
Mg	10.651	6.265	4.944
Mo	0.054	0.044	0.044
P	6.539	5.962	2.999
Zn	1.032	0.065	0.053

Table S7: Phosphorous depletion in wastewater AD2. (Growth conditions: 5:45 inoculum:culture medium ratio, 12:12 h light:dark photoperiod).

Microalga	P (% depletion))
Arthrospira maxima	95.4±0.2
Chlorella vulgaris	8.48±0.02
Isochrysis galbana	99.1±0.2
Nannochloropsis gaditana	65.1±0.1

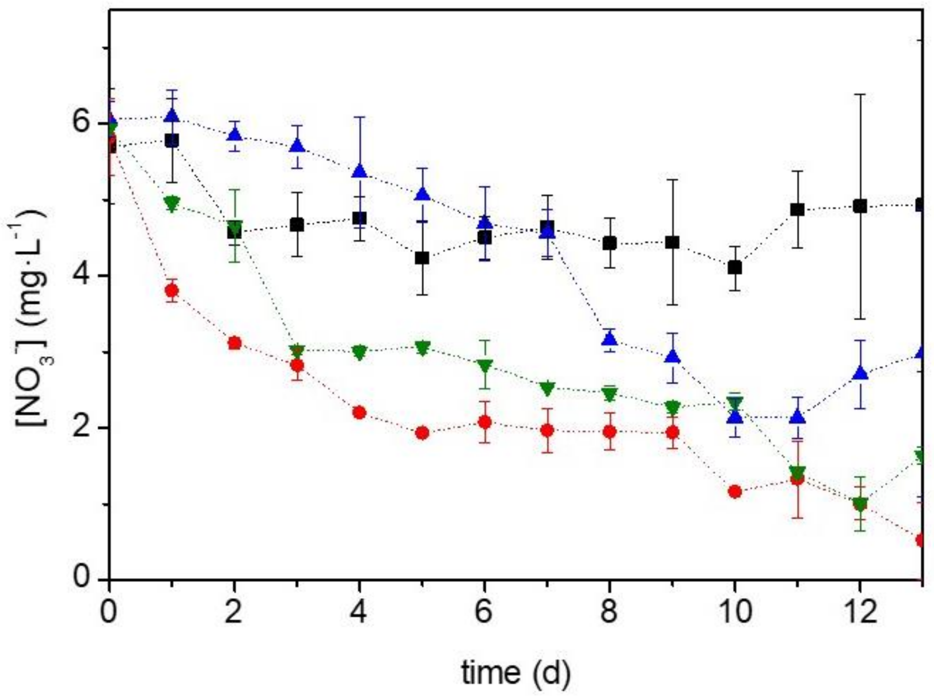

Figure S1: Nitrate uptake in wastewater AD2: ■ *A. maxima*, ● *C. vulgaris*, ▲ *I. galbana* and ▼ *N. gaditana*. (Growth conditions: 5:45 inoculum:culture media ratio, 12:12 h light:dark photoperiod).
